# Biomorphic Engineering of Multifunctional Polylactide Stomatocytes toward Therapeutic Nano‐Red Blood Cells

**DOI:** 10.1002/advs.201801678

**Published:** 2019-01-19

**Authors:** Jingxin Shao, Imke A. B. Pijpers, Shoupeng Cao, David S. Williams, Xuehai Yan, Junbai Li, Loai K. E. A. Abdelmohsen, Jan C. M. van Hest

**Affiliations:** ^1^ Bio‐Organic Chemistry Institute for Complex Molecular Systems Eindhoven University of Technology Helix, het Kranenveld (STO 3.41), P. O. Box 513 5600 MB Eindhoven The Netherlands; ^2^ Department of Chemistry College of Science Swansea University Swansea SA2 8PP UK; ^3^ State Key Laboratory of Biochemical Engineering Institute of Process Engineering Chinese Academy of Sciences Beijing 100190 P. R. China; ^4^ Beijing National Laboratory for Molecular Sciences (BNLMs) CAS Key Lab of Colloid, Interface and Chemical Thermodynamics Institute of Chemistry Chinese Academy of Sciences Beijing 100190 P. R. China

**Keywords:** biomorphic engineering, cell‐mimetic, erythrocytes, hypoxia, stomatocytes

## Abstract

Morphologically discrete nanoarchitectures, which mimic the structural complexity of biological systems, are an increasingly popular design paradigm in the development of new nanomedical technologies. Herein, engineered polymeric stomatocytes are presented as a structural and functional mimic of red blood cells (RBCs) with multifunctional therapeutic features. Stomatocytes, comprising biodegradable poly(ethylene glycol)‐*block*‐poly(D,L‐lactide), possess an oblate‐like morphology reminiscent of RBCs. This unique dual‐compartmentalized structure is augmented via encapsulation of multifunctional cargo (oxygen‐binding hemoglobin and the photosensitizer chlorin e6). Furthermore, stomatocytes are decorated with a cell membrane isolated from erythrocytes to ensure that the surface characteristics matched those of RBCs. In vivo biodistribution data reveal that both the uncoated and coated nano‐RBCs have long circulation times in mice, with the membrane‐coated ones outperforming the uncoated stomatoctyes. The capacity of nano‐RBCs to transport oxygen and create oxygen radicals upon exposure to light is effectively explored toward photodynamic therapy, using 2D and 3D tumor models; addressing the challenge presented by cancer‐induced hypoxia. The morphological and functional control demonstrated by this synthetic nanosystem, coupled with indications of therapeutic efficacy, constitutes a highly promising platform for future clinical application.

The morphology of functional nanosystems is a key parameter in the design of functional nanosystems that are developed for therapeutic applications, due to the impact of parameters, such as size, shape, and surface chemistry on their performance in a biological context.^1^ Biomorphic engineering is a design strategy that attempts to develop particles with characteristics derived from naturally occurring objects, with the aim to attain structures that function harmoniously in a living environment.^2^ As a biological archetype, red blood cells (RBCs) possess unique morphological features, such as high encapsulation of oxygen‐storage proteins (hemoglobin, Hb) and a discoidal (oblate) form with a nonimmunogenic surface that facilitates sustained circulation of these cells in the cardiovascular system.^3^ Mimicking RBCs is an excellent strategy for developing biomorphic therapeutic nanosystems that will be capable of functioning in vivo without the commonly encountered problems of reduced circulation and toxicity.^4^

The most significant challenge in the development of RBC‐mimetic nanosystems is the engineering of topologically discrete discoidal particles that retain sufficient versatility to enable functionalization. Recently, we have demonstrated that morphological control over the self‐assembly of block copolymeric vesicles (polymersomes) can be achieved through a combination of molecular engineering and controlled hypertonic shock.^5^ With this technology, we can produce nanoscopic, biodegradable poly(ethylene glycol)‐*block*‐poly(D,L‐lactide) (PEG‐PDLLA) polymersomes that possess an, RBC‐mimetic, oblate morphology, known as stomatocytes. Such biodegradable stomatocytes possess a unique dual‐compartmentalized structure that can be exploited in the encapsulation of functional macromolecular cargo, which can be utilized as biomimetic reaction systems. However, developing this platform as a biomorphic nanomedical technology requires careful tailoring in order to realize both (multi)functionalization and true stealth‐like surface modification.

As a widely described therapeutic application in nanomedical technologies, photodynamic therapy (PDT) is a key strategy in the treatment of solid tumors due to its advantageous performance in anticancer therapy.^6^ In contrast to traditional chemotherapy/radiation therapies, PDT is noninvasive, has low accumulated toxicity, negligible drug resistance, and is cost‐effective.^7^ However, there are key limitations in the development of effective PDT therapeutic systems that need to be addressed, such as poor solubility of photosensitizers, localized hypoxia in the tumor microenvironment, and tumor penetration.^8^ The issue of hypoxia is particularly pertinent in the treatment of tumors where oxygen levels drop steeply from the peripheral tissue to the interior and significantly decrease PDT efficiency.^9^ In order to solve this issue, several hemoglobin (Hb)‐based therapeutic systems have been well developed.^10^ Although these carriers have similar function to RBCs, the unique discoidal morphology has remained elusive. With such an unusual shape, RBCs are engineered to efficiently perform their function of oxygen transport within a complex biological environment.^11^ Inspired by the challenge to mimic the morphology of blood cells and harness this for therapeutic applications, we have designed smart compartmentalized nanocarriers capable of supplying O_2_ while delivering photoactive drugs in order to demonstrate proof‐of‐principle application in PDT. To this end, the development of PEG‐PDLLA stomatocytes as nanoscopic RBC mimics (nano‐RBCs) that can act as oxygen carriers for enhanced PDT is an interesting target for the biomorphic engineering of therapeutic nanoparticles.

Exploiting the morphology of PEG‐PDLLA stomatocytes, we demonstrate the coencapsulation of hydrophobic chlorin e6 (Ce6), a photosensitizer with low dark toxicity and highly efficient generation of singlet oxygen,^12^ alongside a reservoir of Hb encased in the inner lumen to create nano‐RBCs for PDT therapy. To provide structural integrity and true stealth character, we adopt a strategy of camouflaging the particles with an erythrocyte‐derived cell membrane.^13^ Wrapping dual functionalized PEG‐PDLLA stomatocytes in a cell membrane allows us to preserve the physicochemical properties of the underlying architecture while inheriting a complex array of proteins, antigens, and other functional moieties, which can facilitate effective integration in a biological context.^14^ In this paper, we therefore present a nanosystem that comprises the fundamental features of RBCs while exploiting them as a potential PDT therapeutic technology, as demonstrated by 2D cell studies and 3D tumor models in vitro.

Biodegradable block copolymers PEG_44_‐PDLLA_120_ and amino‐PEG_44_‐PDLLA_120_ were synthesized by ring‐opening polymerization as described in previous published work.^15^ The reaction progress and the obtained products were characterized by nuclear magnetic resonance spectroscopy (Figure S1, Supporting Information), differential scanning calorimetry, and gel permeation chromatography (Figure S2, Supporting Information). Formation of oblate stomatocytes was conducted in two steps; first, spherical polymersomes were self‐assembled via the solvent switch methodology and then their shape transformation was induced under hypertonic (dialysis) conditions, as illustrated in **Figure** **1**a. Stomatocytes were engineered with a partial positive surface charge in order to facilitate the coating of the particles with an erythrocyte cell membrane, which possesses an overall negative potential. Positively charged polymersomes were therefore assembled from a solution of PEG_44_‐PDLLA_120_ and amino‐PEG_44_‐PDLLA_120_ (9:1 w/w) in tetrahydrofuran (THF)/dioxane (1:4 v/v), to which ultrapure water was added up to 50 vol% at a rate of 1 mL h^−1^ with vigorous stirring. The cloudy suspension of polymersomes was thereafter dialyzed against a NaCl solution of 50 × 10^−3^ m at 5 °C to transform spherical polymersomes into oblate stomatocytes (Figure 1b,c).^5^ The resulting oblate nanostructures bear a striking resemblance to the morphology of RBCs (Figure 1d), albeit on a smaller length scale. The characteristic dual‐compartmental structure of PEG‐PDLLA stomatocytes is clearly evident from cryogenic transmission electron microscopy (cryo‐TEM) images. Dynamic light scattering (DLS) data indicated that the average hydrodynamic size of the resulting nanostructures was 316 nm with a polydispersity index (PDI) of 0.06 (Figure S3, Supporting Information).

**Figure 1:**
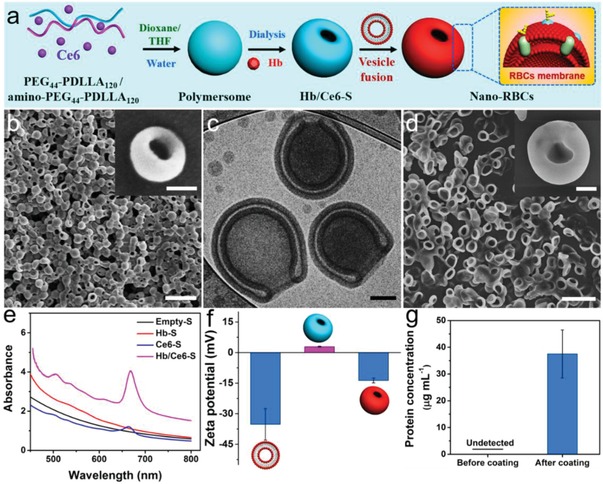
Synthesis and characterization of nano‐RBCs. a) Schematic illustration of the fabrication of biodegradable stomatocytes. b) SEM images of stomatocytes. Scale bar = 1 µm. The inset SEM image is a single stomatocyte with higher magnification. Scale bar = 100 nm. c) Cryo‐TEM image of biodegradable stomatocytes. Scale bar = 100 nm. d) SEM image of RBCs which was isolated from mouse whole blood (Balb/c). Scale bar = 10 µm. The inset is a SEM image of RBCs with higher magnification. Scale bar = 1 µm. e) UV spectrum of different stomatocyte samples (Empty‐S, Hb‐S, Ce6‐S, and Hb/Ce6‐S). f) The surface zeta potential (mV) of RBC vesicles, stomatocytes before and after coating with RBC membranes measured by DLS. g) Protein concentration of stomatocyte samples before and after coating with the RBC membrane vesicles, measured by a BCA protein assay.

In order to construct nano‐RBCs that mimic the physiological function of RBCs while also possessing the necessary photosensitizer for PDT, both Hb and Ce6 were coencapsulated in PEG‐PDLLA stomatocytes. Empty stomatocytes (Empty‐S), Hb‐loaded stomatocytes (Hb‐S), Ce6‐loaded stomatocytes (Ce6‐S), and Hb/Ce6 co‐loaded stomatocytes (Hb/Ce6‐S) were prepared, with visible color changes indicative of successful functionalization (Figure S4, Supporting Information). Ce6 was loaded by dissolving it together with block polymer, and the encapsulation of Hb was achieved by addition of the protein during the shape change process from spherical vesicles to stomatocytes. UV–vis spectra of nano‐RBC samples were used to quantify the incorporation of both Hb and Ce6 (Figure 1e), with successful loading of Hb also confirmed by sodium dodecyl sulfate‐polyacrylamide gel electrophoresis (Figure S5, Supporting Information). After having constructed loaded stomatocytes, the particle surface was decorated with the extracted cell membrane of erythrocytes following literature protocols.^16^ Briefly, erythrocytes were isolated from the peripheral blood of mice (Balb/c, male) and then processed under hypotonic conditions to yield vesicles.^17^ The morphology of RBC vesicular structures (following processing) was confirmed using SEM (Figure S6, Supporting Information). Camouflaged nano‐RBCs were prepared by incubating stomatocytes and RBC‐derived vesicles under gentle shaking for 4 h. Zeta‐potential measurements confirmed that a charge reversal of the positively charged stomatocytes occurred during this process, leading to coated nano‐RBCs with a zeta potential of ≈–15 mV (Figure 1f). The concomitant increase in detectable protein levels in the stomatocyte sample after membrane coating, measured using the bicinchoninic acid (BCA) assay, further validated the success of this strategy (Figure 1g). A fluorescent dye, which associates with the cell membrane, was used to visualize this coating, which was indeed clearly observable on nano‐RBCs using confocal scanning light microscopy (CLSM) (Figure S7a, Supporting Information). The corresponding morphology and average hydrodynamic size of the resulting nano‐RBCs were also measured, as shown in Figure S7b,c in the Supporting Information. Via biomorphic engineering, we were able to fabricate nanoparticles that mimic red blood cells in shape, surface properties, and loading (Hb), with further synthetic functionalization using a photosensitizer.

To investigate the activity of the photosensitizer, the generation of singlet oxygen (^1^O_2_) was detected using a chemical method, whereby 9,10‐anthracenediyl‐bis(methylene)dimalonic acid (ABDA) was employed to react with reactive oxygen species (**Figure** **2**a).^18^ ABDA consumption (and thereby ^1^O_2_ production) was monitored by UV–vis spectroscopy (400 nm). Empty control samples (Empty‐S) showed no ^1^O_2_ generation and ABDA absorbance was stable under laser irradiation (Figure 2b). In contrast, samples containing Ce6 only (Ce6‐S) showed a decreasing ABDA signal indicative of ^1^O_2_ generation, which was even more the case in samples with both Hb and Ce6 present (Hb/Ce6‐S) (Figure 2c,d). Coencapsulated Hb/Ce6‐S outperformed Ce6‐S samples by ≈40%, which can be attributed to the local reservoir of oxygen supplied by Hb (Figure 2e,f). Deoxygenation of the nano‐RBCs solution showed that the performance advantage of the Hb/Ce6‐S system could be annulled in anoxic conditions; however, upon reoxygenation the activity was recovered (Figure 2g–i).

**Figure 2:**
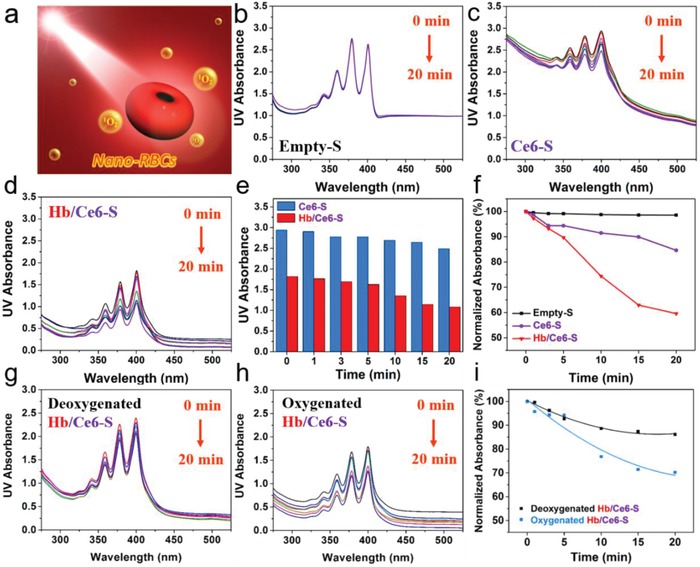
Evaluation of singlet oxygen generation. a) Schematic illustration of the detection of ^1^O_2_ by measuring the changes in UV–vis spectra upon oxidation of ABDA under 660 nm laser irradiation. The time‐lapsed absorption spectra of ABDA in the presence of b) Empty‐S, c) Ce6‐S, and d) Hb/Ce6‐S. All stomatocytes are coated with an erythrocyte cell membrane. e) Difference in ABDA oxidation as a function of time between Ce6‐S and Hb/Ce6‐S. f) Normalized absorbance changes, including a negative control (Empty‐S) and Ce6‐S, together with Hb/Ce6‐S. Absorbance spectra of ABDA in the presence of g) deoxygenated Hb/Ce6‐S, h) oxygenated Hb/Ce6‐S, and i) normalized absorbance spectra.

The beneficial effect of the cell membrane modification of nano‐RBCs was evaluated using mouse macrophage cells (RAW 264.7), measuring relative uptake before (Figure S8a, Supporting Information) and after coating (Figure S8b, Supporting Information). After incubation with RAW 264.7 cells, the cell uptake efficiency was evaluated as a function of time (0, 6, and 12 h) by measuring the fluorescent signal derived from Ce6 using flow cytometry. Compared to naked stomatocytes (14.6%), only 1.8% of coated nano‐RBCs were captured by cells after 12 h incubation. This result indicates that nano‐RBCs inherit the unique antiphagocytic properties of RBCs with significantly suppressed cellular uptake; a highly promising feature for a long‐circulating delivery system that could be applied to cancer therapy. In order to further investigate the systematic circulation time of nano‐RBCs, in vivo fluorescence imaging was conducted. Cyanine 7 (Cy7) was used as a near infrared fluorescent dye to label the nano‐RBCs. Ex vivo fluorescence imaging demonstrated that nano‐RBCs were successfully labeled with Cy7 (Figure S9a, Supporting Information). BALB/c nude mice were divided randomly into three groups, to compare the performance of nano‐RBCs, uncoated stomatocytes and phosphate‐buffered saline (PBS). For each group, 200 µL of sample was injected intravenously through the tail vein and monitored at different time points **Figure** **3**. The fluorescence intensity of the injected particles was furthermore quantitatively analyzed (Figure S9b, Supporting Information) It was observed that both uncoated stomtocytes and nano‐RBCs had long circulation times in vivo, with the coated ones outperforming the uncoated stomatocytes, in particular during the first 30 min after injection. This demonstrates the benefits of both shape and surface characteristics for circulation of nanoparticles.

**Figure 3:**
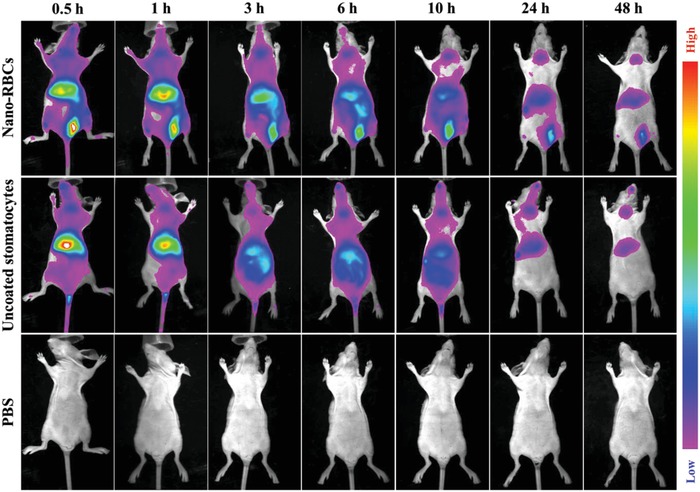
Systematic circulation time of nano‐RBCs evaluated by in vivo fluorescence imaging at designated time points after intravenous injection.

The toxicity of nano‐RBCs toward healthy (NIH/3T3) and cancerous (HeLa) cell lines was evaluated using an 3‐(4,5‐dimethylthiazol‐2‐yl)‐2,5‐diphenyltetrazolium bromide (MTT) assay. Indeed, low dark toxicity was observed in cell membrane coated stomatocytes up to concentrations of 0.25 mg mL^−1^ (Figures S10 and S11, Supporting Information). Cytotoxicity evaluation of nano‐RBCs before cell membrane coating was also conducted (Figure S12, Supporting Information), which demonstrated that the block polymer used was biocompatible. In vitro PDT efficacy of nano‐RBCs was evaluated using HeLa cells. After 6 h, cellular uptake was maximal (Figure S13, Supporting Information) and intracellular localization of nano‐RBCs was confirmed by CLSM (Figure S14, Supporting Information). Compared to macrophage cells (RAW 264.7, Figure S8, Supporting Information), the intracellular content of nano‐RBCs was much higher in HeLa cells. This is not unexpected as for cancerous cells nutrient acquisition, and thus endocytosis, is urgently needed to meet the bioenergetic, biosynthetic, and redox demands.^19^ The cellular uptake toward the nano‐RBCs was also investigated using different types of cell lines; namely, NIH/3T3 (healthy cells) and HepG2 (cancerous cells). The cellular uptake efficiency was 32% for NIH/3T3 cells and 97% for HepG2 cells (Figure S15, Supporting Information).

PDT‐induced cytotoxicity was investigated using a 660 nm laser (0.1 W cm^−2^, 5 min) by measuring apoptosis of HeLa cells (Calcein‐AM as live stain, alongside propidium iodide (PI) for dead cells) (**Figure** **4**). Under control conditions (cells only), or using laser irradiation without Ce6 present, no cellular apoptosis was observed; however, in the presence of either Ce6‐S or Hb/Ce6‐S significant apoptosis was induced after irradiation. Again, the dual functional nano‐RBCs were most effective and displayed enhanced PDT (Figure S16, Supporting Information).

**Figure 4:**
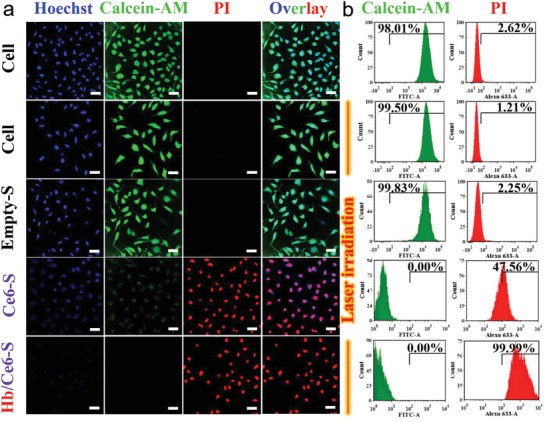
In vitro experiments testing PDT efficacy. a) CLSM images of live/dead assay costained HeLa cells after different treatments (from top to bottom: cells only, cells with laser irradiation, Empty‐S with laser irradiation, Ce6‐S with laser irradiation, and Hb/Ce6‐S with laser irradiation. A 660 nm laser (0.1 W cm^−2^) was used to irradiate the different groups for 5 min. Scale bar = 50 µm. b) Flow cytometry of HeLa cells stained with Calcein‐AM alongside PI for detecting live and dead cells, respectively.

In order to test the PDT efficacy of the nano‐RBCs under hypoxic conditions, a multicellular spheroid (MCS) was used as 3D tumor model.^20^ As 3D tumor models better resemble the tumor microenvironment, they have been widely used to study tumor biology and for screening cancer therapeutics, reducing the burden on animal testing.^21^ Consequently, MCS comprising fibroblasts (NIH/3T3) and breast cancer cells (4T1) were created by coculturing the two cell lines in a 5:1 ratio to prepare a 3D tumor model as good match for clinical tumor stroma.^22^ Alongside two control groups (no treatment and laser irradiation) the remaining MSCs were treated with Empty‐S, Ce6‐S, and Hb/Ce6‐S samples for 6 h, followed by laser irradiation (**Figure** **5**). In line with previous data, control groups did not show any apoptosis, highlighting the inherent biocompatibility of the PEG‐PDDLA stomatocytes. Both Ce6‐S and Hb/Ce6‐S samples showed successful PDT; again this effect was more pronounced in nano‐RBCs with an internal reservoir of O_2_. These results confirm that nano‐RBCs are capable of improving PDT efficacy through their capacity to function as an oxygen carrier.

**Figure 5:**
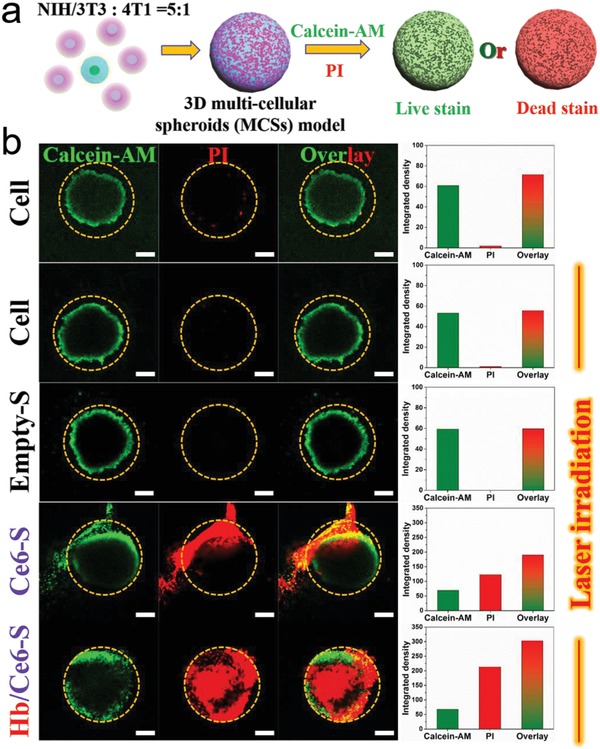
Cell viability under PDT treatment into 3D MCS models as a function of different therapeutic groups. a) Schematic representation of the evaluation of PDT treatment using live/dead fluorescent assay. b) From top to bottom, CLSM images of 3D MCSs without irradiation (control group) and with laser irradiation in the presence of Empty‐S, Ce6‐S, and Hb/Ce6‐S, respectively. Scale bar = 200 µm. The images on the right row were the corresponding integrated fluorescence intensities of each channel (Calcein‐AM, PI, and overlay) which were analyzed by ImageJ.

Through biomorphic engineering, active PDT therapeutic nanoparticles were created that mimicked red blood cells in shape, surface properties, oxygen carrier capability, and long circulation in vivo. Dual compartmentalized, biodegradable PEG‐PDLLA stomatocytes, with a shape reminiscent of erythrocytes, were loaded with hemoglobin and the photosensitizer Ce6. By coating the surface of these particles with the cell membrane of red blood cells, they were given stealth characteristics that shielded them from uptake by macrophages. Systematic circulation tests revealed that such nano‐RBCs displayed a long retention time in vivo. Coencapsulation of Hb and Ce6 led to improved PDT performance of nano‐RBCs both in regular cell culture and a 3D tumor cell model, providing a mechanism to overcome the kind of hypoxia that would be encountered in the tumor microenvironment. Based on these observations we think that nano‐RBCs constitute a highly promising therapeutic platform for future clinical applications.

## Conflict of Interest

The authors declare no conflict of interest.

## Supporting information

Supplementary

## References

[1] a) R. A. Meyer , J. C. Sunshine , J. J. Green , Trends Biotechnol. 2015, 33, 514; 26277289 10.1016/j.tibtech.2015.07.001 PMC4568838

[2] a) J. Cosmidis , A. S. Templeton , Nat. Commun. 2016, 7, 12812; 27628108 10.1038/ncomms12812 PMC5027620

[3] a) N. Mohandas , P. G. Gallagher , Blood 2008, 112, 3939; 18988878 10.1182/blood-2008-07-161166 PMC2582001

[4] a) M. J. Xuan , J. X. Shao , J. Zhao , Q. Li , L. R. Dai , J. B. Li , Angew. Chem., Int. Ed. 2018, 57, 6049; 10.1002/anie.201712996 29480962

[5] a) L. K. E. A. Abdelmohsen , D. S. Williams , J. Pille , S. G. Ozel , R. S. M. Rikken , D. A. Wilson , J. C. M. van Hest , J. Am. Chem. Soc. 2016, 138, 9353; 27374777 10.1021/jacs.6b03984 PMC4974604

[6] a) S. B. Brown , E. A. Brown , I. Walker , Lancet Oncol. 2004, 5, 497; 15288239 10.1016/S1470-2045(04)01529-3

[7] Y. Ma , X. Y. Li , A. J. Li , P. Yang , C. Y. Zhang , B. Tang , Angew. Chem., Int. Ed. 2017, 56, 13752. 10.1002/anie.201708005 28856780

[8] a) J. L. Sandell , T. C. Zhu , J. Biophotonics 2011, 4, 773; 22167862 10.1002/jbio.201100062 PMC3321368

[9] a) J. Zhou , T. Schmid , S. Schnitzer , B. Brüne , Cancer Lett. 2006, 237, 10; 16002209 10.1016/j.canlet.2005.05.028

[10] a) Y. Jia , Y. Cui , J. B. Fei , M. C. Du , L. R. Dai , J. B. Li , Y. Yang , Adv. Funct. Mater. 2012, 22, 1446;

[11] a) D. E. Discher , N. Mohandas , E. A. Evans , Science 1994, 266, 1032; 7973655 10.1126/science.7973655

[12] a) X. X. Tan , X. J. Pang , M. Z. Lei , M. Ma , F. Guo , J. P. Wang , M. Yu , F. P. Tan , N. Li , Int. J. Pharm. 2016, 503, 220; 26988376 10.1016/j.ijpharm.2016.03.019

[13] a) S. W. Tan , T. T. Wu , D. Zhang , Z. P. Zhang , Theranostics 2015, 5, 863; 26000058 10.7150/thno.11852 PMC4440443

[14] a) Y. H. Zhai , J. H. Su , W. Ran , P. C. Zhang , Q. Yin , Z. W. Zhang , H. J. Yu , Y. P. Li , Theranostics 2017, 7, 2575; 28819448 10.7150/thno.20118 PMC5558554

[15] B. G. G. Lohmeijer , R. C. Pratt , F. Leibfarth , J. W. Logan , D. A. Long , A. P. Dove , F. Nederberg , J. Choi , C. Wade , R. M. Waymouth , J. L. Hedrick , Macromolecules 2006, 39, 8574.

[16] a) J. G. Piao , L. M. Wang , F. Gao , Y. Z. You , Y. J. Xiong , L. H. Yang , ACS Nano 2014, 8, 10414; 25286086 10.1021/nn503779d

[17] a) Q. Jiang , Z. M. Luo , Y. Z. Men , P. Yang , H. B. Peng , R. R. Guo , Y. Tian , Z. Q. Pang , W. L. Yang , Biomaterials 2017, 143, 29; 28756194 10.1016/j.biomaterials.2017.07.027

[18] a) S. Kim , T. Y. Ohulchanskyy , H. E. Pudavar , R. K. Pandey , P. N. Prasad , J. Am. Chem. Soc. 2007, 129, 2669; 17288423 10.1021/ja0680257 PMC2556058

[19] a) D. Hanahan , R. A. Weinberg , Cell 2011, 144, 646; 21376230 10.1016/j.cell.2011.02.013

[20] a) C. Fischbach , R. Chen , T. Matsumoto , T. Schmelzle , J. S. Brugge , P. J. Polverini , D. J. Mooney , Nat. Methods 2007, 4, 855; 17767164 10.1038/nmeth1085

[21] L. Su , R. C. Li , S. Khan , R. Clanton , F. W. Zhang , Y. N. Lin , Y. Song , H. Wang , J. W. Fan , S. Hernandez , A. S. Butters , G. Akabani , R. MacLoughlin , J. Smolen , K. L. Wooley , J. Am. Chem. Soc. 2018, 140, 1438. 29350522 10.1021/jacs.7b11462

[22] a) D. L. Priwitaningrum , J. G. Blondé , A. Sridhar , J. van Baarlen , W. E. Hennink , G. Storm , S. L. Gac , J. Prakash , J. Controlled Release 2016, 244, 257; 10.1016/j.jconrel.2016.09.004 27616660

