# Supplementary material for: Biomorphic Engineering of Multifunctional Polylactide Stomatocytes toward Therapeutic Nano‐Red Blood Cells
Source: Adv Sci (Weinh). 2019 Jan 19;6(5):1801678. doi: 10.1002/advs.201801678 (PMC6402394; doi:10.1002/advs.201801678)
Supplement: Supplementary file 1 — Supplementary [file ADVS-6-1801678-s001.pdf]

Copyright WILEY-VCH Verlag GmbH & Co. KGaA, 69469 Weinheim, Germany, 2019.

ADVANCED SCIENCE
Open Access

Supporting Information

for *Adv. Sci.,* DOI: 10.1002/advs.201801678

Biomorphic Engineering of Multifunctional Polylactide Stomatocytes toward Therapeutic Nano-Red Blood Cells

Jingxin Shao, Imke A. B. Pijpers, Shoupeng Cao, David S. Williams, Xuehai Yan, Junbai Li, Loai K. E. A. Abdelmohsen, and Jan C. M.van Hest**

Copyright WILEY-VCH Verlag GmbH & Co. KGaA, 69469 Weinheim, Germany, 2019.

Supporting Information

Biomorphic Engineering of Multifunctional Polylactide Stomatocytes towards Therapeutic Nano-Red Blood Cells

Jingxin Shao, Imke A. B. Pijpers, Shoupeng Cao, David S. Williams, Xuehai Yan, Junbai Li, Loai K. E. A. Abdelmohsen, and Jan C. M. van Hest**

Contents

1 Materials and Methods

1.1 Materials

1.2 Methods

1.2.1 Dynamic light scattering measurements (DLS)

1.2.2 Scanning electron microscopy (SEM)

1.2.3 Cryogenic transmission electron microscopy (Cryo-TEM)

1.2.4 Nuclear magnetic resonance spectroscopy (NMR)

1.2.5 Differential scanning calorimetry (DSC)

1.2.6 Gel permeation chromatography (GPC)

1.2.7 Confocal laser scanning microscopy (CLSM)

1.2.8 UV-vis spectroscopy

1.2.9 Flow cytometry

1.2.10 Microplate reader

1.2.11 In-Vivo imaging system

1.2.12 Synthesis of poly(ethylene glycol)$_{44}$-poly(D,L-Lactide)$_{120}$ (PEG_{44}-$PDLLA_{120}$) and amino-PEG_{44}-$PDLLA_{120}$ block polymers

1.2.13 Fabrication of stomatocytes and loading with hemoglobin (Hb)/Chlorin e6 (Ce6)

1.2.14 Preparation and characterization of RBC vesicles, and RBC-derived vesicle modified stomatocytes

1.2.15 Evaluation of the generation of singlet oxygen ($^1O_2$)

1.2.16 Cell culture

1.2.17 Uptake efficacy

1.2.18 *In vivo* fluorescence imaging

1.2.19 Cell viability

1.2.20 *In vitro* evaluation of photodynamic therapy (PDT)

1.2.21 3D multi-cellular spheroid (MCS) tumor model

2 Results

Figure S1. ^1H-NMR spectrum of copolymers.

Figure S2. GPC trace of polymers.

Figure S3. Size distribution of biodegradable stomatocytes.

Figure S4. Optical images of stomatocyte samples.

Figure S5. SDS-PAGE protein analysis.

Figure S6. SEM images of red blood cell membrane vesicles.

Figure S7. Characterization of nano-RBCs by CLSM, SEM, and DLS.

Figure S8. Endocytosis rate.

Figure S9. *Ex-vivo* and *in vivo* fluorescence imaging.

Figure S10. Cell viability of NIH/3T3 cells.

Figure S11. Cell viability of HeLa cells.

Figure S12. Cell viability of Hb/Ce6-S before coating with cell membrane.

Figure S13. Cellular uptake of nano-RBCs as a function of time.

Figure S14. CLSM image of intracellular localization.

Figure S15. Cellular uptake of nano-RBCs towards NIH/3T3 and HepG2.

Figure S16. Cell viability of HeLa cells after different treatments.

3 References

1 Materials and Methods

1.1 Materials

Poly(ethylene glycol) (PEG) 2K was purchased from JenKem technology. D,L-lactide was purchased from Acros Organics. Sodium chloride (NaCl) was obtained from Merck. Dialysis Membrane MWCO 12-14000 g mol^{-1} from Spectra/Pro® was used for dialysis during stomatocytes formation. Chlorin e6 (Ce6) was purchased from Frontier Scientific. Dulbecco's modified eagle medium (DMEM), RPMI 1640 medium, Trypsin-EDTA, Penicillin streptomycin, no mycoplasma fetal bovine serum (FBS), Hank's buffered salt solution (HBSS), phosphate buffered saline (PBS), protein bicinchoninic acid (BCA) protein assay kit, Calcein-AM, propidium iodide (PI), Hoechst 33342, and Alexa Fluor™ 488 conjugate of wheat germ agglutinin were purchased from ThermoFisher Scientific. Mini-PROTEAN@ TGX Stain-Free™ Gels, 4 × Laemmli protein sample buffer and Precision Plus Protein™ All Blue Standards were obtained from Bio-Rad. Cyanine 7 (Cy7) was purchased from Lumiprobe GmbH. All other chemicals were supplied by Sigma-Aldrich. The Fusion 100 syringe pumps were purchased from Chemyx Inc. Ultrapure Milli Q (Millipore) water (18.2 MΩ·cm) was used for all experiments in this work. All chemicals were used as received without further purification.

1.2 Methods

1.2.1 Dynamic light scattering measurements (DLS)

DLS measurements were performed by using a Malvern Instruments Zetasizer (model Nano ZSP). Zetasizer software was used to process and analyze the data.

1.2.2 Scanning electron microscopy (SEM)

Morphology of stomatocytes was characterized by SEM (FEI Quanta 200 3D FEG).

1.2.3 Cryogenic transmission electron microscopy (Cryo-TEM)

Experiments were performed by using cryo-TEM (FEI Tecnai G2 Sphere, 300 kV electron source) equipped with LaB6 filament equipped with autoloader station.

1.2.4 Nuclear magnetic resonance spectroscopy (NMR)

Proton nuclear magnetic resonance measurements were performed on a Bruker 400 Ultrashield™ spectrometer equipped with a Bruker SampleCase autosampler, using $CDCl_3$ as a solvent and TMS as internal standard.

1.2.5 Differential scanning calorimetry (DSC)

DSC measurements were conducted using a TA Instruments Multicell DSC. By scanning from -20 °C up to 80 °C at 5 °C per minute, the *Tg* value was recorded from the second heating run.

1.2.6 Gel permeation chromatography (GPC)

Molecular weights of the block polymer were determined by using a Prominence GPC system (Shimadzu) with a PL gel 5 µm mixed D column (Polymer Laboratories) and a differential refractive index detector. THF was used as an eluent with a flow rate of 1 mL per minute. Polystyrene strandards (580~377400 g mol^{-1}) were used for calibration.

1.2.7 Confocal laser scanning microscopy (CLSM)

Fluorescence images were observed and captured by using CLSM (Zeiss LSM510 META NLO, and Leica TCS SP5X).

1.2.8 UV-vis spectroscopy

Drug loading efficiency, BCA protein assay, as well as the generation of singlet oxygen were characterized by UV-vis spectroscopy (V-650, JASCO).

1.2.9 Flow cytometry

Cell uptake and cell viability were measured by flow cytometry (BD Biosciences, USA).

1.2.10 Microplate reader

Cell viability was evaluated *via* a standard 3-(4,5-dimethylthiazol-2-yl)-2,5-diphenyl tetrazolium bromide (MTT) assay by using a microplate reader (Safire^2, TECAN).

1.2.11 In-Vivo imaging systems

Biodistribution of nano-RBCs *in vivo* was measured by In-Vivo imaging systems (FX Pro, KODAK) at designed time points.

1.2.12 Synthesis of poly(ethylene glycol)$_{44}$-poly(D,L-Lactide)$_{120}$ (PEG$_{44}$-PDLLA$_{120}$) and amino-PEG$_{44}$-PDLLA$_{120}$ block polymers

According to published protocols, PEG$_{44}$-PDLLA$_{120}$ block polymers were synthesized by ring-opening polymerization (ROP) starting from PEG-macro initiators using the organic base 1,8-diazabicycloundec-7-ene (DBU) as a catalyst.[1] The reaction progress and the final product were analyzed by ^{1}H-NMR spectroscopy. For the synthesis of amino-PEG$_{44}$-PDLLA$_{120}$, a 50 mL round bottom flask was dried with a heat gun and flushed with argon. Boc-NH-PEG-OH (2 kDa, 200 mg, 0.1 mmol) was weighed in together with D,L-lactide (12 mmol, 1.73 gr), dissolved in dry toluene and concentrated in vacuo to remove excess water. Then, dry DCM (20 mL) was added to the reagents and the solution was stirred under argon until all materials were dissolved. Next, DBU (0.05 mmol, 8

μL) was added and the reaction was stirred under argon for two hours at room temperature. The disappearance of monomer peaks was confirmed with ^{1}H-NMR. The reaction mixture was diluted with DCM, extracted with $KHSO_4$ (2x), water and brine. The solution was dried with Na_2SO_4, filtered and concentrated in vacuo yielding a yellowish oil. To remove the Boc protective group, the polymer was dissolved in 5 mL of 4 M HCl in dioxane for 1 hour. The reaction mixture was concentrated in vacuo, dissolved in dioxane and freeze-dried, yielding a white powder (83%). The obtained amphiphilic polymer was characterized using DSC and SEC. For PEG_{44}-$PDLLA_{120}$ and amino-PEG_{44}-$PDLLA_{120}$, the *Tg* value was 21 ^oC, and the PDI was 1.07. The relative M_ns compared to PS standards obtained from GPC were 29.6 and 19.8 kg mol^{-1}, respectively. All the products were stored at -20 ^oC under argon until use.

1.2.13 Fabrication of stomatocytes and loading with hemoglobin (Hb)/Chlorin e6 (Ce6)

PEG_{44}-$PDLLA_{120}$ and amino-PEG_{44}-$PDLLA_{120}$ (9:1 w/w, 20 mg) were dissolved in 2 mL of mixed organic solvent (THF : dioxane = 1:4 v/v) in a 15 mL vial. Then a magnetic stirring bar was added to the solution and the vial was sealed with a rubber septum. The mixed solution was stirred for at least 30 min before adding 2 mL of ultrapure MilliQ water *via* a syringe pump (1 mL h^{-1}). Afterwards, the resulting cloudy solution was transferred into a prehydrated dialysis bag (12-14 kDa, 2 mL cm^{-1}) and dialyzed against a pre-cooled NaCl solution (50 mM) for 24 h at 5 ^oC with a NaCl solution change after 1 h. The morphology of the stomatocytes was characterized by SEM, cryo-TEM, and DLS. For loading the stomatocytes with Ce6, 1 mg Ce6 was dissolved together with the block polymers (20 mg) in 2 mL of THF and dioxane (1:4 v/v). For loading with Hb, 6 mg mL^{-1} Hb aqueous solution (2 mL) was added to the cloudy solution before dialysis against salt solution. The loading efficacy of Ce6 (640 nm) and Hb (628 nm) was measured by UV-vis spectroscopy.[2] The drug loading efficiency (DLE) is defined as the ratio of weight of drug loaded to the weight of drug in the feed.[3] The DLE of Ce6 and Hb were 44.76% and 97.50%, respectively. Furthermore, Hb loading was characterized by SDS-PAGE.[4]

1.2.14 Preparation and characterization of RBC vesicles, and RBC-derived vesicle modified stomatocytes

The RBCs were isolated from peripheral blood of healthy mice (Balb/c, male) following published protocols.[5] Briefly, blood was centrifuged for 5 min (800×g, 4^oC) and then washed three times with ice cold PBS buffer (pH 7.4, 1×). Hemolysis was obtained by adding the hypotonic solution and then storing the above-mentioned solution overnight at 4 ^oC. After that, the blood samples were treated on an ice bath for 20 min, followed by washing with PBS buffer and sonication. The obtained RBC-derived vesicles were stored at 4 ^oC for further use. The RBCs and RBC-derived vesicles were characterized by SEM and CLSM. Erythrocyte membrane coated stomatocytes were prepared according to a published method.[5] Stomatocytes were mixed with the RBC-derived vesicles and co-

cultured for 4 h under gentle shaking at 4 ^{o}C. Free RBC-derived vesicles were removed by centrifugation at 10000 rpm for 1 min. For observation by CLSM, RBC-derived vesicles and erythrocyte membrane coated stomatocytes were stained with the Alexa Fluor^{TM} 488 conjugate of wheat germ agglutinin. The surface charge changes of stomatocytes before and after coating were measured by Zetasizer. Surface protein content of stomatocytes before and after coating with the erythrocyte membrane were measured by a BCA protein assay kit. Additionally, the yield of membrane coating process was measured by flow cytometry, which is 91.51%. Here, the fluorescent signal from Ce6 was used to count the whole number of stomatocytes, whilst cell membrane fluorescent dye (Alexa Fluor^{TM} 488 conjugate of wheat germ agglutinin) was detected to count the membrane coating stomatocytes.

1.2.15 Evaluation of the generation of singlet oxygen ($^1O_2$)

9,10-Anthracenediyl-bis(methylene)dimalonic acid (ABDA) was used as an indicator to assess the generation of $^1O_2$, according to a chemical oxidation method.[6] Stomatocytes (1.5 mL, 0.25 mg mL^{-1}) were homogeneously mixed with ABDA/DMSO solution (25 μL, 4 mg mL^{-1}), followed by illumination with a 660 nm laser (BeamQ Lasers). The absorbance intensity of ABDA at 400 nm as a function of time was recorded by UV-Vis spectroscopy.

1.2.16 Cell culture

Mice embryonic fibroblast cells (NIH/3T3), human cervical cancer cells (HeLa), murine breast carcinoma cell line (4T1), liver hepatocellular carcinoma (HepG2) and mice macrophage cells (RAW 264.7) were cultured in cell culture medium supplemented with 10% FBS and 1% penicillin-streptomycin at 37 ^{o}C in the cell incubator (ThermoFisher Scientific) with an atmosphere of 5% CO_2 and 70% humidity. For NIH/3T3, HeLa, and 4T1, DMEM was used as cell culture medium. For RAW 264.7, RPMI 1640 medium was used.

1.2.17 Uptake efficacy

The uptake efficacy of stomatocytes before and after coating with the erythrocyte membrane towards RAW 264.7 was measured by flow cytometry. Briefly, nano-RBCs (0.25 mg mL^{-1}) were incubated with RAW 264.7 for 0 h, 6 h, and 12 h. Non-internalized stomatocytes were removed through washing the cells with PBS three times. The fluorescent signal was detected by flow cytometry. The same method was user to determine the cellular uptake of HeLa, NIH/3T3 and HepG2 towards the nano-RBCs.

1.2.18 *In vivo* fluorescence imaging

Female BALB/c nude mice (6 weeks, body weight ~18 g) were obtained by Department of Experimental Animals, Institute of Process Engineering, Chinese Academy of Sciences (Beijing, China).

After acclimatization for 5 days, the mice were randomaly divided into three groups, and injected intravenously through the tail vein of 200 µL nano-RBCs, and uncoated stomatocytes (1 mg mL^{-1}, 1×PBS, pH 7.4). The third group as control group was injected 200 µL PBS (1×, pH 7.4). Here, as near infrared fluorescent dye, Cy7 was loaded into the stomatocytes for *in vivo* fluorescence imaging. The biodistribution of nano-RBCs was observed by In-Vivo imaging system at designed time points. All animal experiments were conducted under the guidelines and approved by the local ethics committe.

1.2.19 Cell viability

NIH/3T3 and HeLa cells were used for cytotoxicity studies. Cells were diverted to 96-well plates using a standard trypsin-based technique with a final concentration of 5×10^4 cells per mL. When the cell density reached 90%, different samples at a range of concentrations (0, 0.05, 0.1, 0.15, 0.2, and 0.25 mg mL^{-1}) were added. Then a standard 3-(4,5-dimethylthiazol-2-yl)-2,5-diphenyl tetrazolium bromide (MTT) assay was used to evaluate cell viability in the presence of the different samples.

1.2.20 *In vitro* evaluation of photodynamic therapy (PDT)

HeLa cells were seeded in µ-Slide 8 wells (Ibidi) and cultured in DMEM cell culture medium containing 10% FBS and 1% penicillin-streptomycin at 37 ^{o}C, 5% CO_2 and 70% humidity. To optimize the culture time, stomatocyte samples were cultured with cells for 0 h, 2 h, 4 h, and 6 h, and then measured by CLSM (Leica TCS SP5X) and flow cytometry. To evaluate the therapeutic efficacy, 0.25 mg mL^{-1} samples were co-cultured with HeLa cells for 6 h, followed by irradiation with a 660 nm laser (0.1 W cm^{-1}) for 5 min, after washing the cells with HBSS to remove stomatocytes that were not taken up by the cells. The cells were then cultured for another 12 h in the cell incubator, and analyzed by CLSM after live/dead fluorescent staining. The cell viability after PDT treatment was quantified with an MTT assay and by flow cytometry.

1.2.21 3D multi-cellular spheroid (MCS) tumor model

3D MCS tumor models were produced by co-culturing NIH/3T3 and 4T1 (5:1) cells, according to previously published protocols with slight modifications.[7] Cells were seeded in agarose coated 96-well plates. 0.15 g of agarose was added to 10 mL of low glucose DMEM cell culture medium (1.5% wt/vol) in an appropriate beaker, followed by sealing with an aluminum foil/lid. After autoclaving (120 ^{o}C, 20 min), the agarose solution was transferred to a 96-wells plate (flat bottomed, 50 µL/well) under sterile conditions. Then a concave surface was formed by the solidification of the agarose. Next, NIH/3T3 and 4T1 cells with a ratio of 5:1 (200 µL, 6×10^4 cells mL^{-1} in high glucose DMEM cell culture medium) were co-seeded in the above-mentioned 96-wells plate, and were then cultured in the incubator with an atmosphere of 5% CO_2 and 70% humidity for 4 days for the formation of MCSs.

For evaluation of the PDT efficacy, MCSs were divided into five groups and cultured with different stomatocyte samples for 6 h. Cold PBS was then used to wash the MCS three times. Subsequently, the MCSs were irradiated with a 660 nm laser for 5 min (660 nm, 0.1 W cm^{-2}) and cultured for another 12 h. Calcein-AM and PI were used to co-stain the MSCs for assessing the cell viability with CLSM. The corresponding integrated fluorescent intensity of each channel was further analyzed by ImageJ.

2 Results

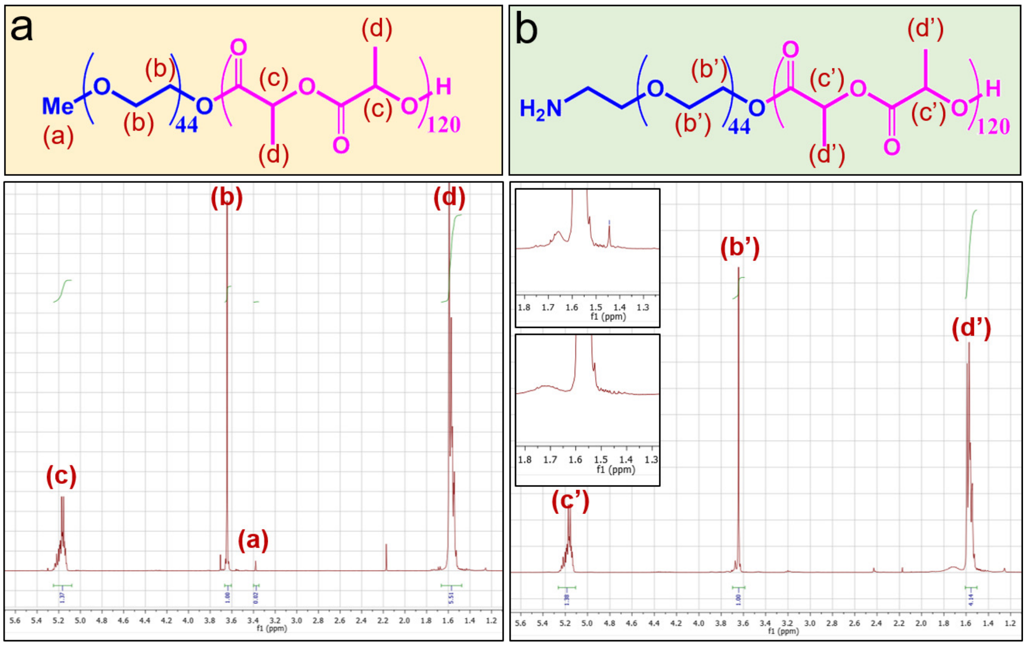

Figure S1. ^{1}H-NMR spectrum of copolymers. a) PEG_{44}-$PDLLA_{120}$. b) Boc deprotected H_2N-PEG_{44}-$PDLLA_{120}$. The Boc protection group (singlet 1.41 ppm) has disappeared (see inset).

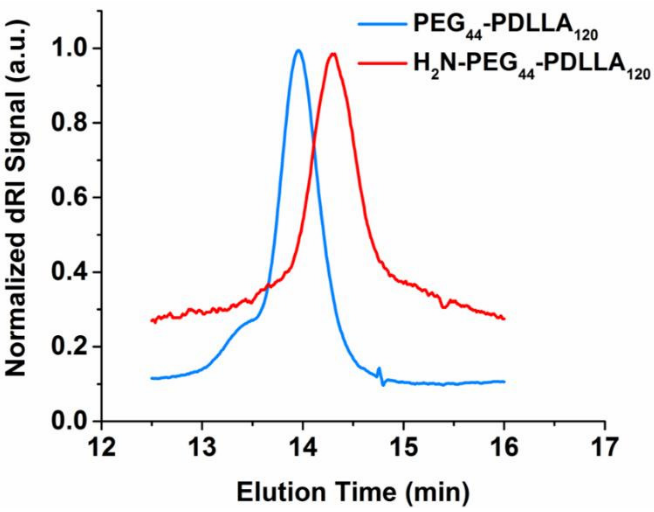

Figure S2: GPC trace of polymers PEG_{44}-$PDLLA_{120}$ (blue) and amine functionalized H_2N-PEG_{44}-$PDLLA_{120}$ (red). Mn values relative to PS standards were 29.6 and 19.8 kg mol^{-1} respectively.

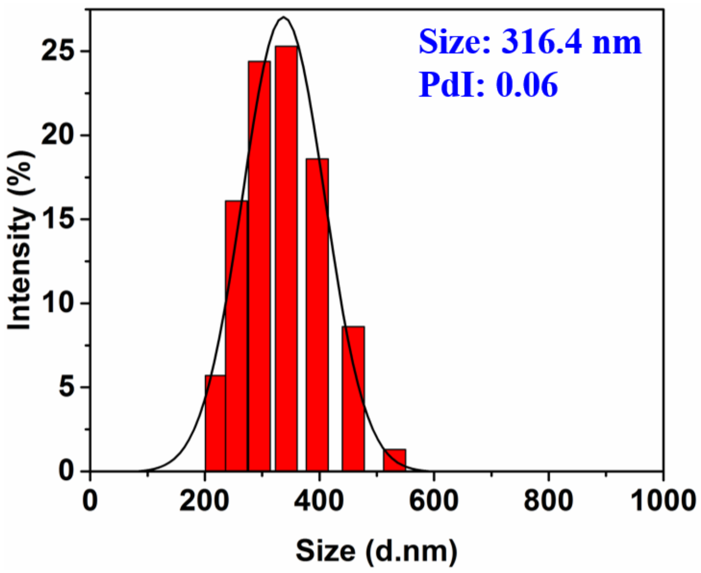

Figure S3. Size distribution of biodegradable stomatocytes measured by dynamic light scattering.

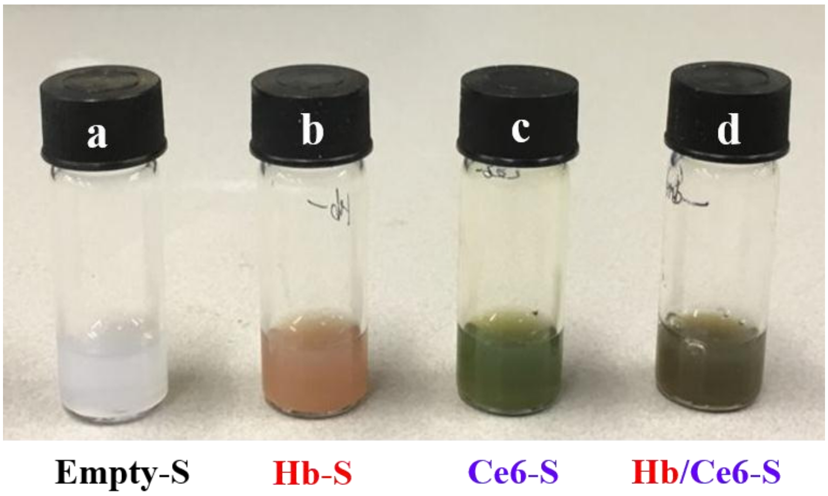

Figure S4. Optical images of stomatocyte samples. (a) Empty stomatocytes (Empty-S). (b) Hemoglobin loaded stomatocytes (Hb-S). (c) Chlorin e6 loaded stomatocytes (Ce6-S). (d) Hb and Ce6 loaded stomatocytes (Hb/Ce6-S).

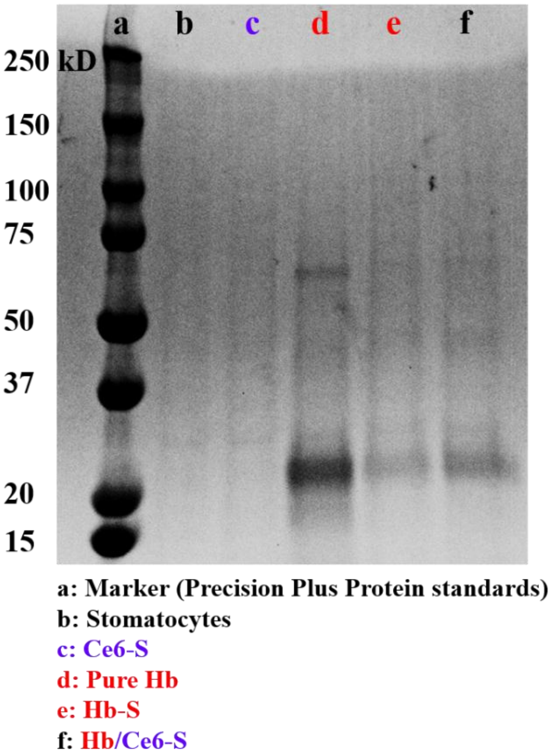

Figure S5. SDS-PAGE protein analysis indicating that Hb was successfully loaded into Hb and Hb/Ce6 stomatocytes (Hb-S, Hb/Ce6-S). Based on the Hb standard solution (6 mg mL^{-1}, channel d), the Hb concentration in Hb/Ce6-S (channel f) was obtained by analysis of gel band intensities using ImageJ, which is 4.57 mg mL^{-1}.

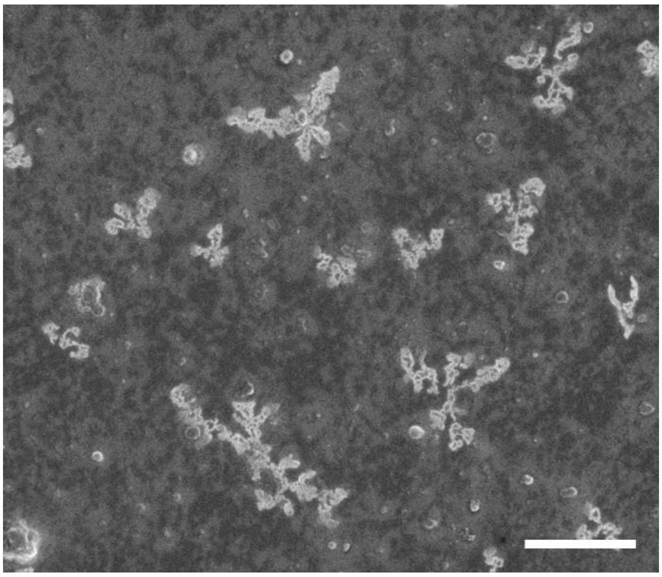

Figure S6. SEM image of red blood cell membrane vesicles (Scale bar = 2 μm).

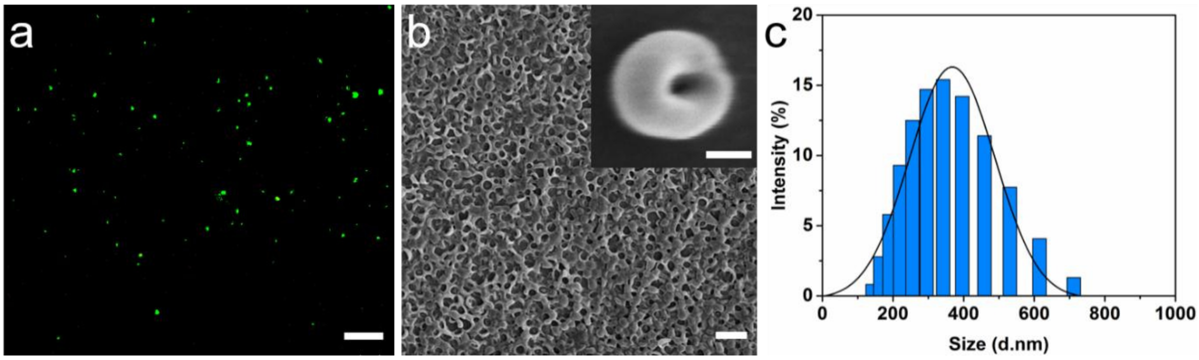

Figure S7. Characterization of nano-RBCs. (a) CLSM image of nano-RBCs. Green fluorescence corresponds to the cell membrane dye (Wheat Germ Agglutinin, Alexa Fluor ™ 488 Conjugate). Scale bar = 20 μm. (b) SEM image of nano-RBCs. Scale bar = 1 μm. Insert image is the single nano-RBCs with higher magnification. Scale bar = 100 nm. (c) The average hydrodynamic size of the resulting nano-RBCs.

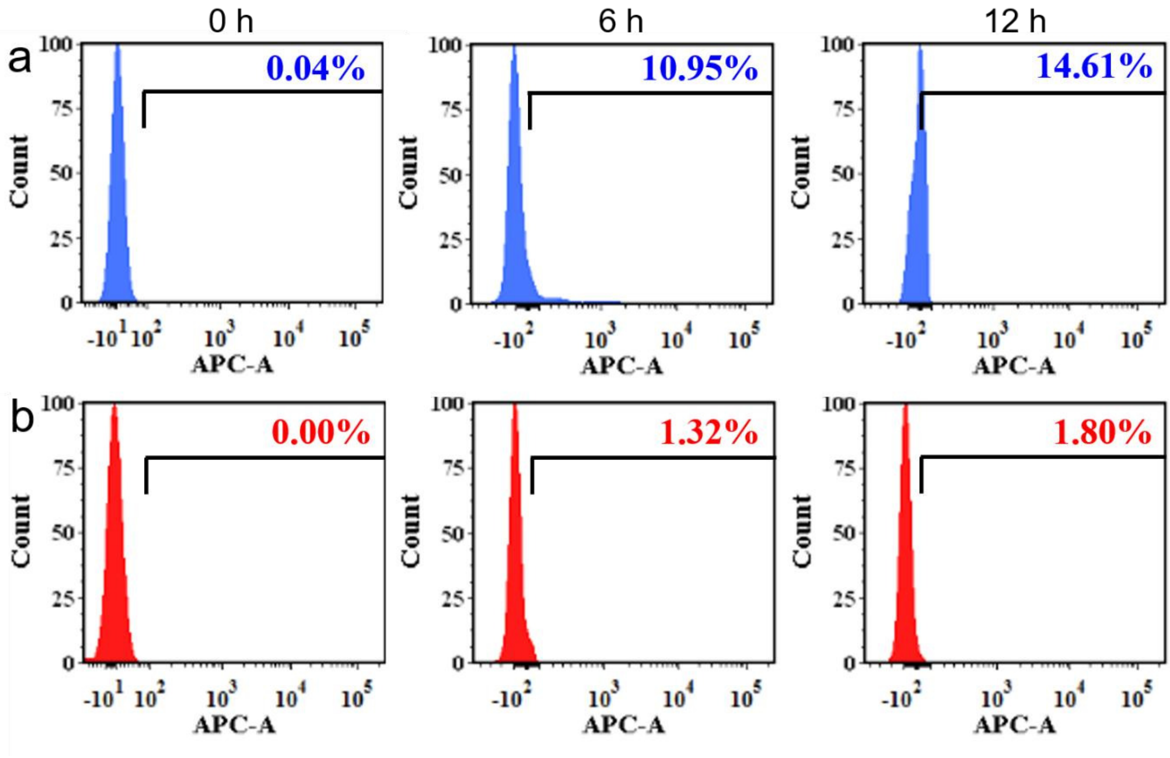

Figure S8. Endocytosis rate analyzed by flow cytometry. Hb/Ce6-S before (a) and after (b) coating with the erythrocyte cell membrane by macrophage cells (RAW 264.7) at 0 h, 6 h, and 12 h, respectively.

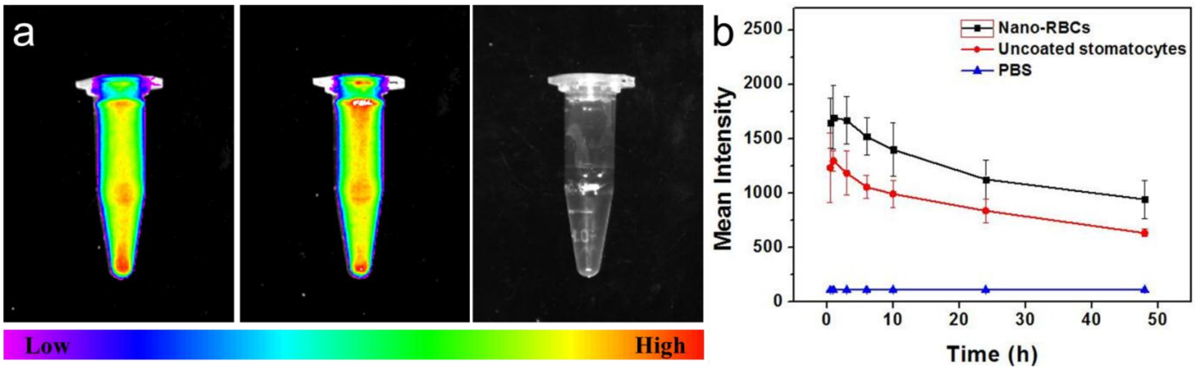

Figure S9. (a) *Ex-vivo* fluorescence imaging of nano-RBCs (left), uncoated stomatocytes (middle) and PBS (right). (b) Quantified fluorescence intensity from each group at different time intervals were analysed by imaging software (Carestream MI).

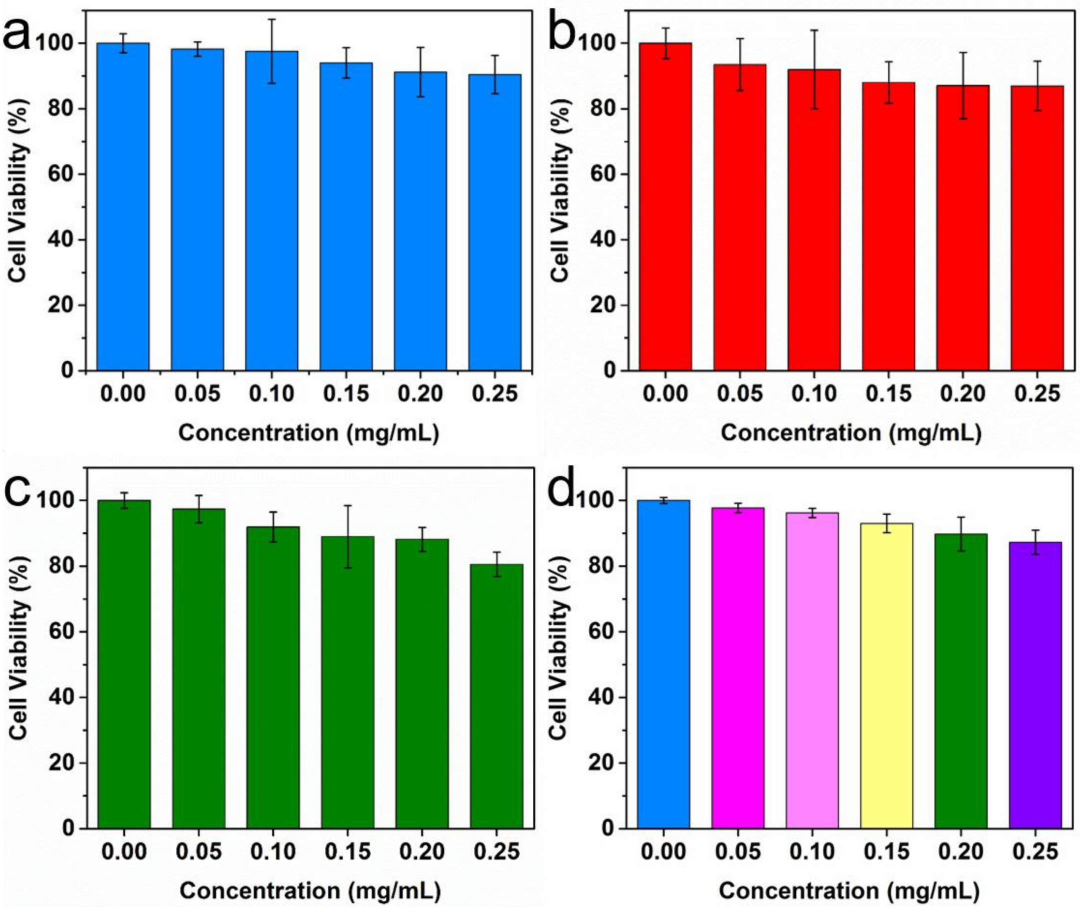

Figure S10. Cytotoxicity evaluation of Empty-S (a), Hb-S (b), Ce6-S (c), and Hb/Ce6-S (d) by measuring the cell viability of mouse embryonic fibroblast cells (NIH/3T3) through the standard MTT assay.

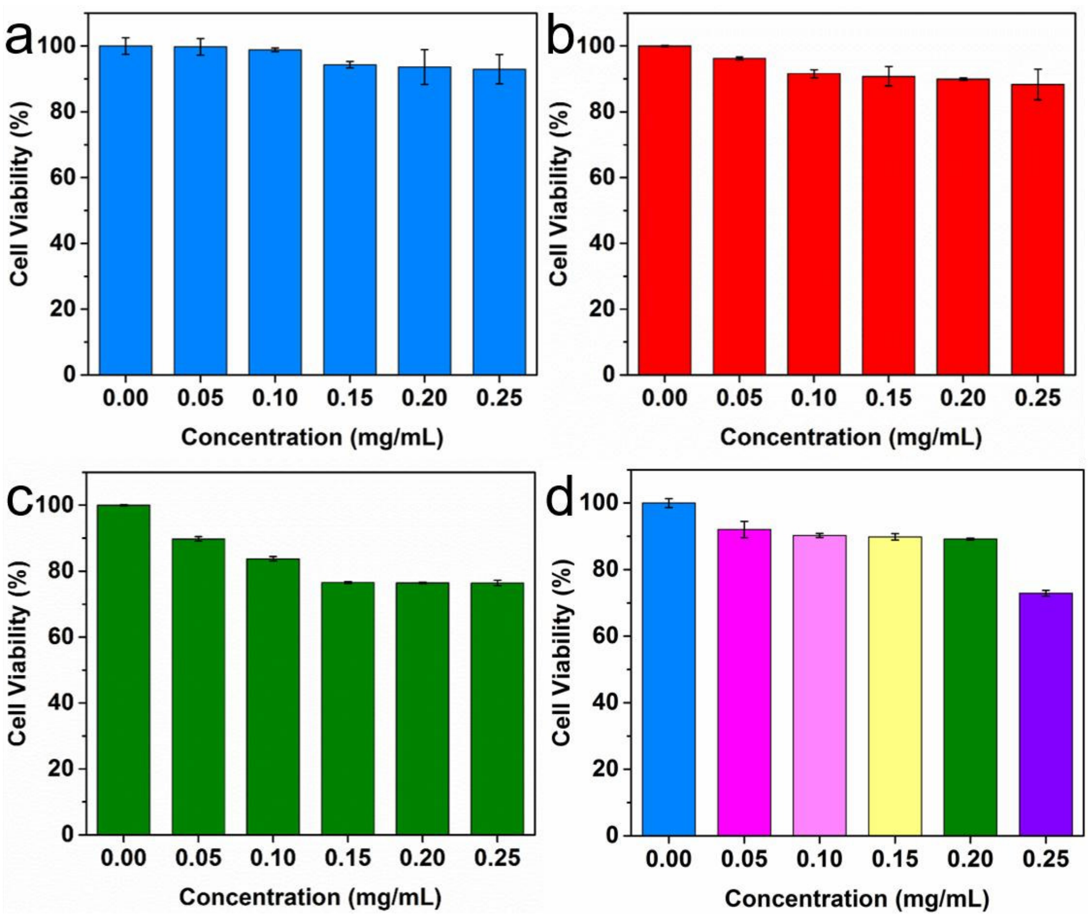

Figure S11. Cytotoxicity evaluation of Empty-S (a), Hb-S (b), Ce6-S (c), and Hb/Ce6-S (d) by measuring the cell viability of human cervical cancer cells (HeLa) through the standard MTT assay.

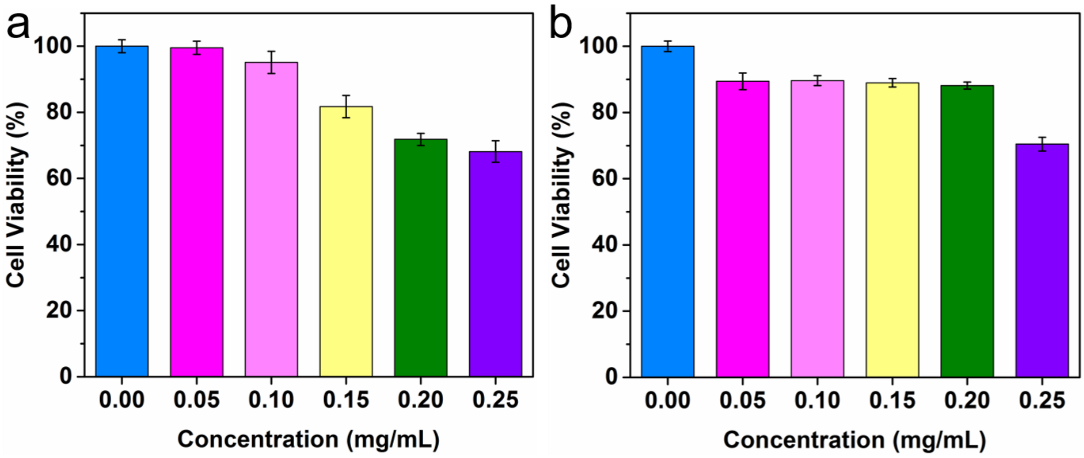

Figure S12. Cytotoxicity evaluation of Hb/Ce6-S before coating with cell membrane by measuring the cell viability of NIH/3T3 (a) and HeLa (b) through the standard MTT assay.

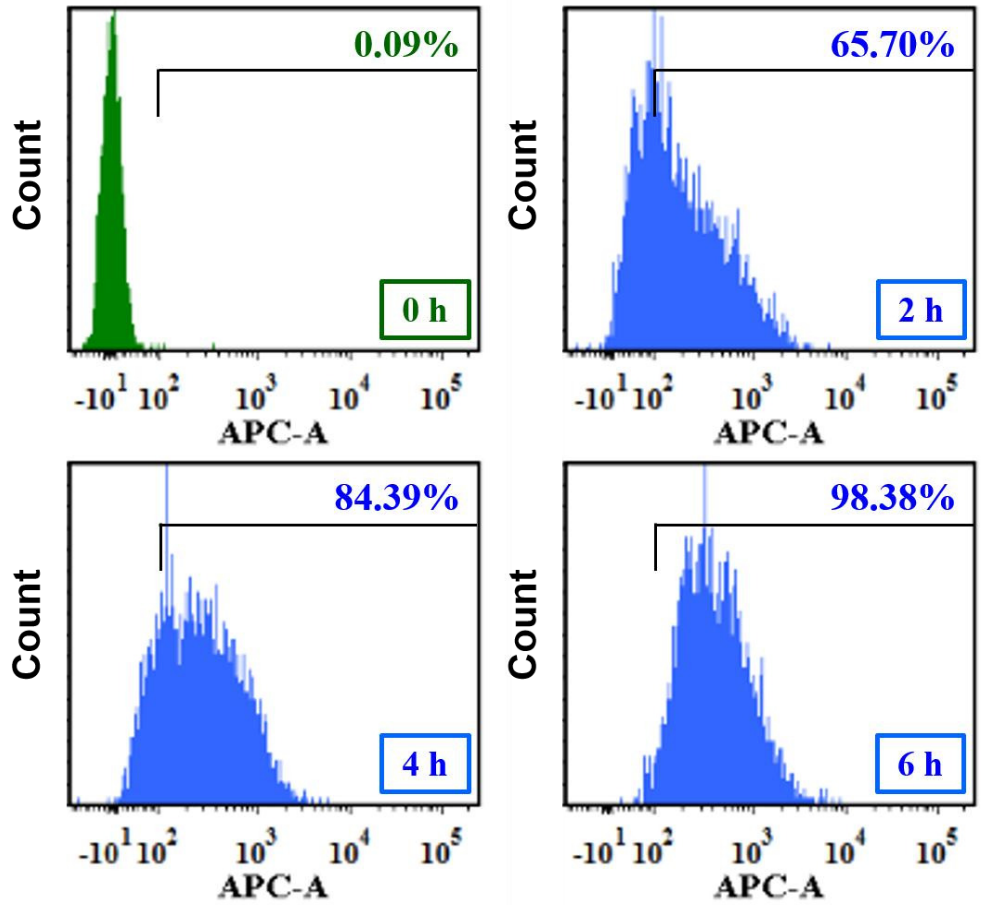

Figure S13. Cellular uptake of nano-RBCs as a function of time (0 h, 2 h, 4 h, and 6 h), measured by flow cytometry. The higher endocytosis rate in HeLa cells compared to macrophage cells (**Figure S8**) can be explained by their need for nutrient acquisition to meet their bioenergetics, biosynthetic, and redox demands.[8]

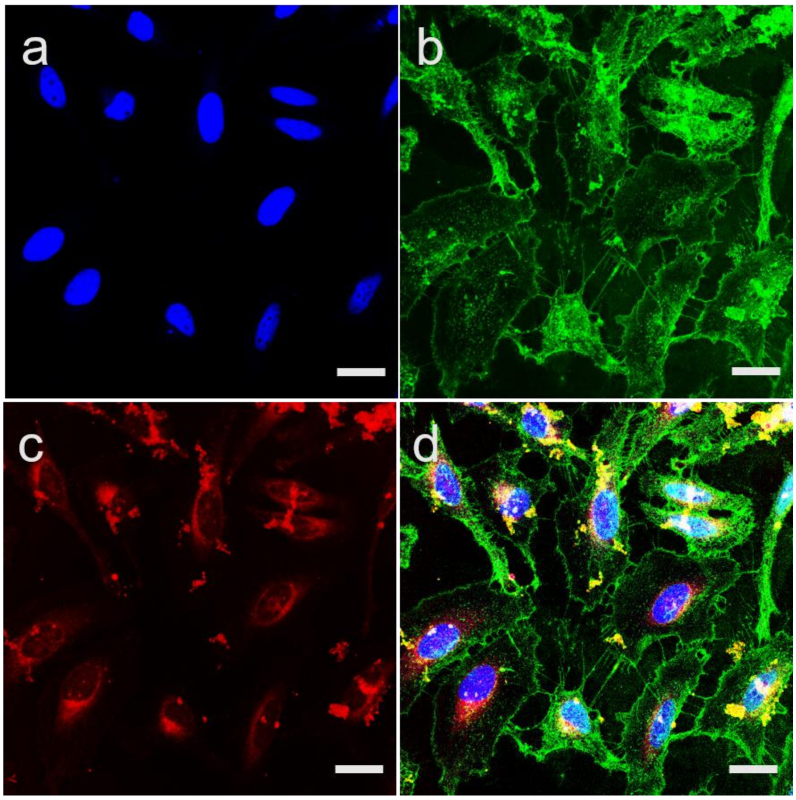

Figure S14. CLSM images of intracellular localization of nano-RBCs (0.25 mg mL^{-1}) in HeLa cells by staining cell nucleus with Hoechst 33342 (a) and Alexa Fluor 488 conjugate of wheat germ agglutinin for cell membrane staining (b). (c) Red fluorescent signal was derived from Ce6. (d) Overlay image. Scale bar = 30 μm.

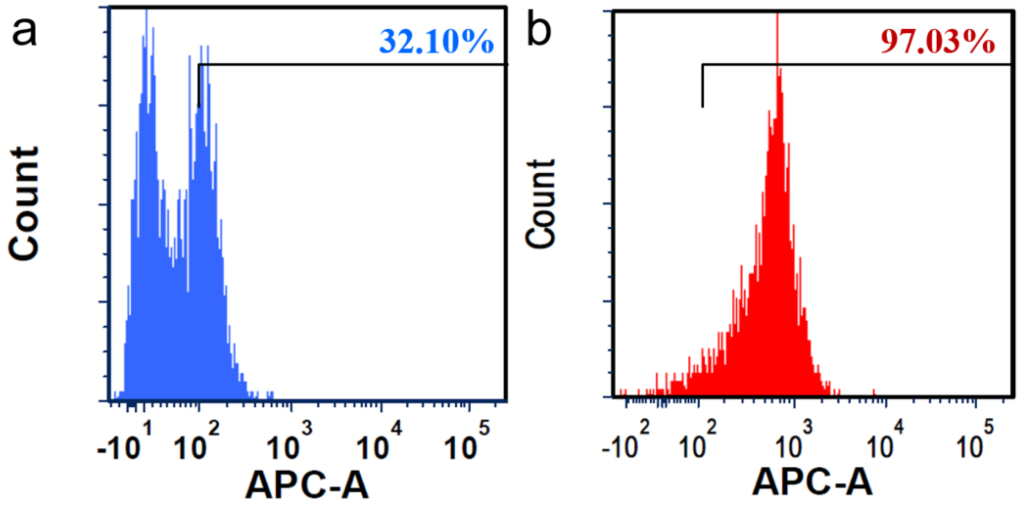

Figure S15. Cellular uptake of nano-RBCs after culturing for 6 h measured by flow cytometry. (a) NIH/3T3. (b) HepG2.

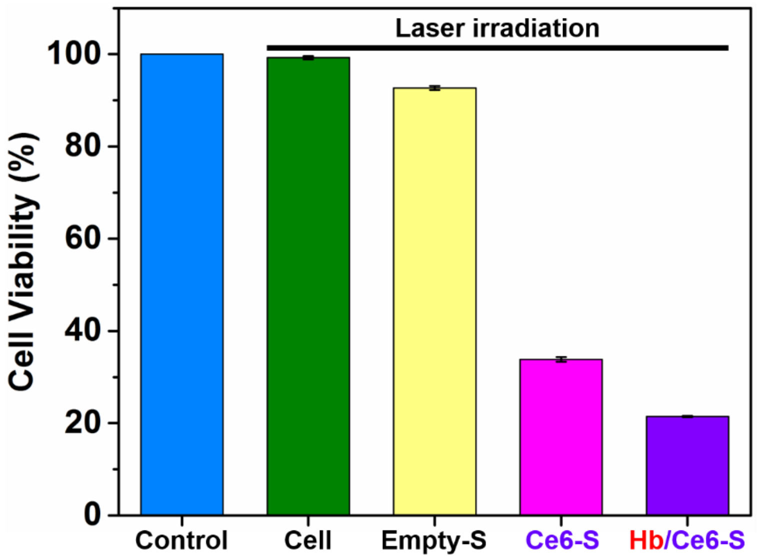

Figure S16. Cell viability of HeLa cells after different treatments (dark control, cells only, Empty-S, Ce6-S, and Hb/Ce6-S) measured by the MTT assay. Compared to other groups, Hb/Ce6-S exhibits best performance in PDT treatment. The experiments were conducted in triplicate.

3 References

[1] a) L. K. E. A. Abdelmohsen, D. S. Williams, J. Pille, S. G. Ozel, R. S. M. Rikken, D. A. Wilson, J. C. M. van Hest, *J. Am. Chem. Soc.* **2016**, *138*, 9353; b) I. A. B. Pijpers, L. K. E. A. Abdelmohsen, D. S. Williams, J. C. M. van Hest, *ACS Macro Lett.* **2017**, *6*, 1217.

[2] a) F. L. Gao, M. Z. Sun, L. G. Xu, L. Q. Liu, H. Kuang, C. L. Xu, *Adv. Funct. Mater.* **2017**, *27*, 1700605; b) W. G. Zijlstra, A. Buursma, *Comp. Biochem. Physiol.* **1997**, *118B*, 743.

[3] C. C. Hung, W. C. Huang, Y. W. Lin, T. W. Yu, H. H. Chen, S. C. Lin, W. H. Chiang, H. C. Chiu, *Theranostics* **2016**, *6*, 302.

[4] Y. Xiong, A. Steffen, K. Andreas, S. Müller, N. Sternberg, R. Georgieva, H. Bäumler, *Biomacromolecules* **2012**, *13*, 3292.

[5] a) J. G. Piao, L. M. Wang, F. Gao, Y. Z. You, Y. J. Xiong, L. H. Yang, *ACS Nano* **2014**, *8*, 10414; b) W. W. Gao, C. M. J. Hu, R. H. Fang, B. T. Luk, J. Su, L. F. Zhang, *Adv. Mater.* **2013**, *25*, 3549; c) C. M. J. Hu, L. Zhang, S. Aryal, C. Cheung, R. H. Fang, L. F. Zhang, *Proc. Natl. Acad. Sci. U. S. A.* **2011**, *108*, 10980.

[6] a) M. J. Xuan, J. X. Shao, J. Zhao, Q. Li, L. R. Dai, J. B. Li, *Angew. Chem. Int. Ed.* **2018**, *57*, 6049; b) H. L. Liu, Y. Yang, A. H. Wang, M. J. Han, W. Cui, J. B. Li, *Adv. Funct. Mater.* **2016**, *26*, 2561.

[7] a) D. L. Priwitaningrum, J. G. Blondé, A. Sridhar, J. van Baarlen, W. E. Hennink, G. Storm, S. L. Gac, J. Prakash, *J. Controlled Release* **2016**, *244*, 257; b) J. Friedrich, C. Seidel, R. Ebner, L. A. Kunz-Schughart, *Nat. Protoc.* **2009**, *4*, 309.

[8] a) D. Hanahan, R. A. Weinberg, *Cell* **2011**, *144*, 646; b) R. J. DeBerardinis, N. S. Chandel, *Sci. Adv.* **2016**, *2*, e1600200; c) N. N. Pavlova, C. B. Thompson, *Cell Metab.* **2016**, *23*, 27.
